# Barriers to penicillin allergy de-labeling in the inpatient and outpatient settings: a qualitative study

**DOI:** 10.1186/s13223-023-00842-y

**Published:** 2023-10-11

**Authors:** Esra Alagoz, Megan Saucke, Prakash Balasubramanian, Paul Lata, Tyler Liebenstein, Sujani Kakumanu

**Affiliations:** 1 Department of Surgery, University of Wisconsin-Madison, Madison, WI USA; 2 William S. Middleton Veterans Memorial Hospital Madison, Madison, WI USA; 3 Department of Medicine, Division of Allergy, Pulmonary and Critical Care, University of Wisconsin-Madison, Madison, WI USA

**Keywords:** Penicillin allergy de-labeling, De-labeling, Antimicrobial stewardship, Inpatient, Outpatient, Qualitative methods, Implementation science

## Abstract

**Background:**

Penicillin allergy is the most commonly reported drug allergy in the US. Despite evidence demonstrating that up to 90% of labels are incorrect, scalable interventions are not well established. As part of a larger mixed methods investigation, we conducted a qualitative study to describe the barriers to implementing a risk-based penicillin de-labeling protocol within a single site Veteran’s hospital.

**Methods:**

We conducted individual and group interviews with multidisciplinary inpatient and outpatient healthcare teams. The interview guides were developed using the Theoretical Domains Framework (TDF) to explore workflows and contextual factors influencing identification and evaluation of patients with penicillin allergy. Three researchers iteratively developed the codebook based on TDF domains and coded the data using thematic analysis.

**Results:**

We interviewed 20 clinicians. Participants included three hospitalists, five inpatient pharmacists, one infectious disease physician, two anti-microbial stewardship pharmacists, four primary care providers, two outpatient pharmacists, two resident physicians, and a nurse case manager for the allergy service. The factors that contributed to barriers to penicillin allergy evaluation and de-labeling were classified under six TDF domains; knowledge, skills, beliefs about capabilities, beliefs about consequences, professional role and identity, and environmental context and resources. Participants from all groups acknowledged the importance of penicillin de-labeling. However, they lacked confidence in their skills to perform the necessary evaluations, such as test dose challenges. The fear of inducing an allergic reaction and adding further complexity to patient care exacerbated their reluctance to de-label patients. The lack of ownership of de-labeling initiative was another significant obstacle in establishing consistent clinical workflows. Additionally, heavy workloads, competing priorities, and ease of access to alternative antibiotics prevented the prioritization of tasks related to de-labeling. Space limitations and nursing staff shortages added to challenges in outpatient settings.

**Conclusion:**

Our findings demonstrated that barriers to penicillin allergy de-labeling fall under multiple behavioral domains. Better role clarification, opportunities to develop necessary skills, and dedicated resources are needed to overcome these barriers. Future interventions will need to employ a systemic approach that addresses each of the behavioral domains influencing penicillin allergy de-labeling with stakeholder engagement of the inpatient and outpatient health care teams.

**Supplementary Information:**

The online version contains supplementary material available at 10.1186/s13223-023-00842-y.

## Background

Penicillin allergy is the most common drug allergy reported by patients in the United States at a rate of 10%. [1] However, several studies have demonstrated that up to 90% of patients who are labeled with penicillin allergy are in fact able to tolerate penicillin [2–4]. The label of penicillin allergy impacts antibiotic prescribing practices, resulting in avoidance of Beta-lactam antibiotics [5], and overuse of broad-spectrum antibiotics, perpetuating the risk of drug-resistant infections [6]. As a result, the mislabeling of penicillin allergy represents a gap in healthcare quality that contributes to unnecessary healthcare costs and increases patient-related complications [7].

Several studies have shown that interventions to remove penicillin allergy labels, commonly referred to, as “de-labeling” patients for penicillin allergy, can be effective. These interventions often cite the need for a multidisciplinary approach with stakeholder engagement from antimicrobial stewardship committees, pharmacists, nurses, and physicians [8]. Studies of de-labeling interventions that have been successful in large patient populations reported that integration of electronic medical record (EMR) tools into clinical workflows such as best practice alerts, along with training of the primary medical team, and patient counseling are needed for long-term success [9]. In many institutions, these essential steps have been supplied by health care staff liaisons who have a particular interest in drug allergy and antimicrobial stewardship. While effective, this dependence on a limited pool of trained personnel can impede wide dissemination and long-term implementation of penicillin allergy de-labeling initiatives. In hospital-based interventions, early identification of patients with penicillin allergy and involvement of the inpatient pharmacy team have been identified as two key factors to pilot study success [10]. The development and use of risk assessment algorithms and point of care tools to de-label patients within a patient encounter have been effective to advance widespread adoption of penicillin allergy de-labeling initiatives [11]. In addition, involvement of a multidisciplinary medical team that is inclusive of antimicrobial stewardship, primary and specialty services is critical to the success of the penicillin allergy de-labeling process. Recent literature has advocated for an increased focus on implementation science to improve penicillin allergy. [12] However, there is paucity of research employing theories of implementation science to determine the barriers and facilitators of successful penicillin allergy de-labeling programs or long-term sustainability and scalability of them.

## Methods

### Current clinic workflow of inpatient penicillin de-labeling

**Fig. 1:**
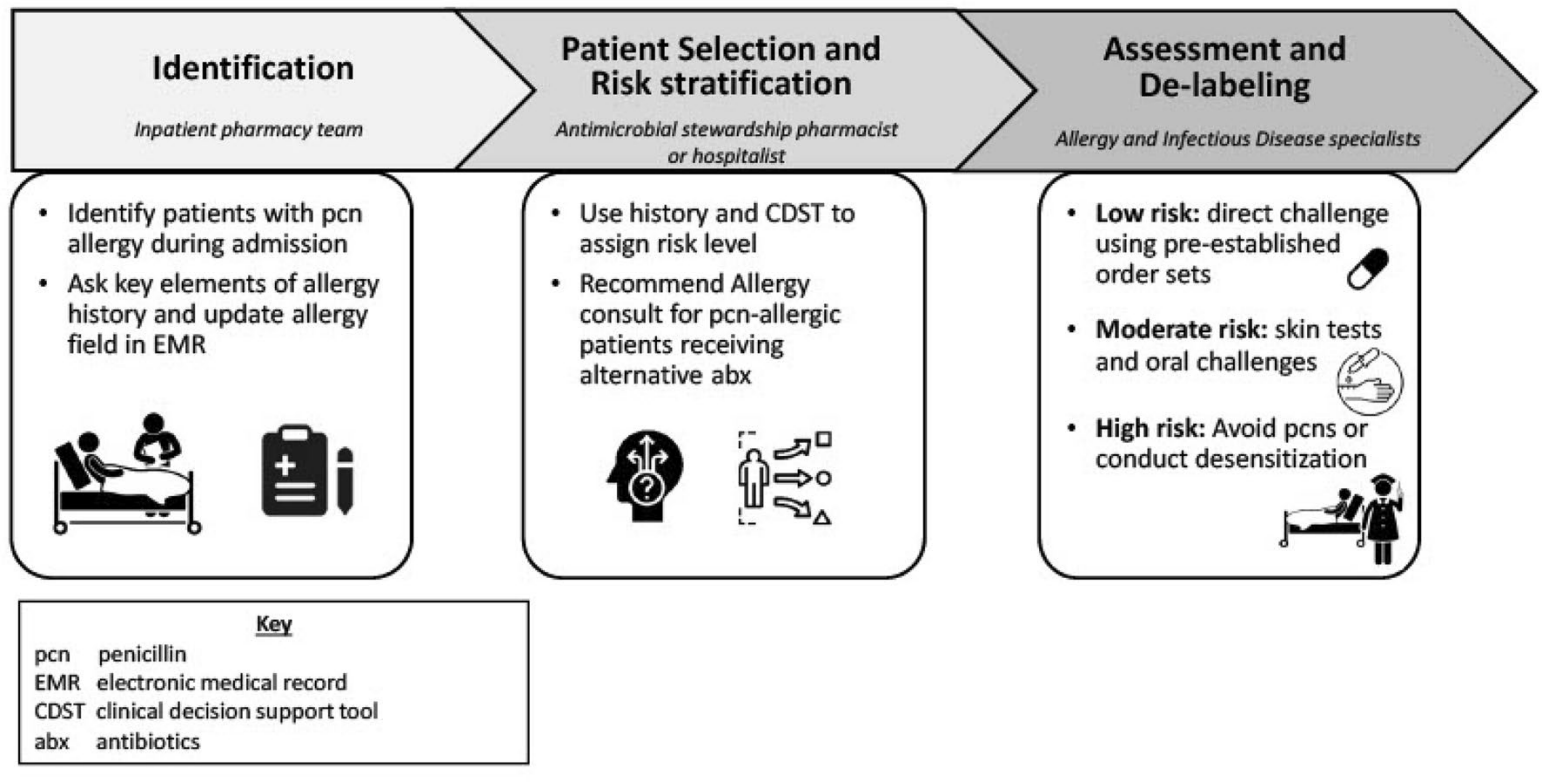
Current state of penicillin allergy de-labeling process for inpatients

Figure 1 describes the current workflow of our *inpatient* setting. When patients are admitted to the hospital, a member of the pharmacy team (i.e. either a pharmacy technician or pharmacist) completes an inpatient medication intake. Penicillin allergy may be entered or reviewed at this time, and if present, will appear as an alert in the patient’s chart when medications are ordered. If the penicillin allergy label affects choices of antibiotic treatment or prevents use of the first-line antibiotic, the inpatient team -including the hospitalists had the option to either follow the algorithm independently or consult the Allergy service if they preferred. The clinical decision-making support tool (CDST) (see Additional files 1, 2, 3, 4: Appendix S1a–d) recommended one of three pathways based on their risk level: *low risk*: an inpatient direct drug challenge to penicillin; *moderate risk*: skin testing followed by drug challenge if skin testing is negative; or high risk: avoidance or inpatient drug desensitization. For low-risk patients, the inpatient team may choose to proceed as directed by the algorithm, without consulting the Allergy service if they were comfortable but also had the option to consult the Allergy service if they were not comfortable with this approach. This current workflow resulted in gaps of care for those who missed screening due to underutilization of the CDST [13, 14]. A multidisciplinary approach needs to be established for sustainable change and adoption.

### Developing and implementing the clinical decision-making support tool (CDST)

As part of an ongoing quality improvement initiative within our VA Hospital, we developed a risk assessment algorithm and an embedded order set which we now call the *CDST* (Additional files 1, 2, 3, 4: Appendix S1a–d). This tool was first developed by modifying the guidelines and algorithms recommended in previous literature [8]. We revised and refined the CDST every 90 days through input from inpatient pharmacists, Infectious Disease (ID) specialists, and hospitalists. Inpatient teams (hospitalists, inpatient pharmacists and nurses) were provided with in person and asynchronous communication regarding the availability of the CDST as well as one didactic session describing how to use the CDST in the inpatient setting. Subsequently, patients who were hospitalized between May–September of 2019 were reviewed. 126 patients with a penicillin allergy label were identified during their inpatient hospital stay. Of these patients, 28% of patients were de-labeled during their hospital stay, and 15% of patients were identified and risk stratified during their hospital stay, but had evaluation deferred by the primary hospital team due to severity of their hospitalized illness. However, as high as 57% of patients were identified as penicillin allergic but missed further screening and evaluation without a clear reason. To delineate the results of this initiative and investigate the barriers to screening the majority of patients, we designed a mixed methods study at our institution. In this paper, we report the qualitative results of our study.

### Theoretical framework

For this study, we used the Theoretical Domains Framework (TDF) to describe the barriers to penicillin de-labeling in inpatient hospital settings as they are perceived by multidisciplinary healthcare teams and patients. Successful implementation of evidence-based interventions in healthcare requires studying and refining the behaviors within the healthcare team [15]. This calls for an integrative theoretical approach that targets multiple behaviors simultaneously. TDF is a conceptual framework that synthesizes 33 well-established theories and 128 key theoretical constructs related to behavior change [16, 17]. The TDF framework comprises 14 domains, each of which includes multiple constructs that further define the foundation of each behavioral domain [18]. Each TDF domain has been linked and mapped onto behavior change techniques [16, 17] to identify and target relevant behaviors in a specific context to ensure successful implementation and uptake of a complex intervention. Although it is widely used in various healthcare settings and practices, applications of TDF to develop interventions for penicillin de-labeling are scarce.

### Design and setting

The research described here is part of a larger study on de-labeling penicillin allergies at a single site Veterans hospital. We defined Penicillin allergy as patients who reported an allergy to penicillin or a related antibiotic (amoxicillin, ampicillin, piperacillin, oxacillin, methicillin, nafcillin). We invited multidisciplinary inpatient and outpatient healthcare teams to participate in our study and conducted one-on-one and group interviews with participants that showed interest between December 2021 and June 2022. We anticipated that these interviews would sensitize us to important dynamics that were not captured by the quantitative data collected for our quality improvement initiative, as semi-structured interviews are ideal for understanding the nuances of participants’ perspectives on barriers to penicillin de-labeling processes. Our study adheres to established criteria for reporting qualitative research [19] (Additional file 5: Appendix S2).

### Data collection

Members of the multidisciplinary healthcare teams were invited via email to participate in one-time interviews between December 2021 and June 2022. We developed semi-structured interview guides using the TDF to explore workflows and contextual factors influencing identification and evaluation of patients with a label of penicillin allergy and their clinical workflow. The questions were also informed by clinical experience and knowledge of general workflows (Table 1). We pilot tested the interview questions with two antimicrobial stewardship pharmacists and an infectious disease physician before data collection commenced. We refined the guide iteratively as interviews progressed to tailor questions to different positions on the multidisciplinary teams and to improve question clarity.

**Table 1:** Codebook, code definitions, and related interview questions

TDF Domain (*definition*)	Constructs	Selected Interview Question(s)
Knowledge *(An awareness of the existence of something)*	Knowledge Procedural knowledge	• How do you think patients will benefit from de-labeling? • What are the key questions to ask when taking a history for patients with pcn allergy? • Once you take the clinical history, do you know the next steps of evaluating a patient with pcn allergy?
Skills *(An ability or proficiency acquired through practice)*	Skills Practice	• Have you ever evaluated patients with pcn allergy? • How often do you evaluate patients with pcn allergy?
Beliefs about capabilities *(Acceptance of the truth, reality or validity about an ability, talent or facility that a person can put to constructive use)*	Perceived competence	• How comfortable are you in determining a patient’s risk of future reaction? • How comfortable are you administering an oral drug challenge to a patient determined to be low risk?
Beliefs about consequences *(Acceptance of the truth, reality, or validity about outcomes of a behavior in a given situation)*	Outcome expectancies	• What fears do you have about the consequences of recommending de-labeling (for patients)? • What fears do you have about the consequences of recommending de-labeling (for the healthcare team)?
Professional role and identity *(A coherent set of behaviors and displayed personal qualities of an individual in a work setting)*	Professional role and identity	• Which services take the lead on pcn allergy de-labeling? • What does the workflow among the services look like?
Environmental context and resources *(Any circumstance of a person’s situation or environment that discourages or encourages the development of skills and abilities, independence, and adaptive behavior)*	Organizational culture or climate Environmental stressors Resources or material resources	• How do communications [or communication gaps] between specialty services and primary services influence the de-labeling process? • How much does de-labeling have priority among your other clinical responsibilities? • What do you think is the most significant barrier to incorporating de-labeling into your work? • Since our allergy resources are limited, what do you think the system can manage without allergy’s involvement? • What technological constraints impede querying the record and documenting what you find?

*pcn* penicillin

A trained, masters-level qualitative interviewer (MS) conducted the interviews virtually using the HIPAA-compliant Microsoft WebEx platform. Interviews lasted 30–60 min. We requested that participants describe their knowledge of penicillin de-labeling and perceived barriers to incorporating it into their clinical workflow. We asked follow-up questions and probes based on participant responses. The Webex conferences were audio recorded, transcribed, de-identified, and imported into NVivo 12 (QSR International) for data management and analysis.

### Ethical considerations

An Institutional Review Board approved the study and granted minimal risk status. Participants were provided with written information about the study, told participation was voluntary, and given the opportunity to ask questions. Identifying information was removed from transcripts to ensure confidentiality. All participants provided written consent for participation.

### Data analysis

Three researchers analyzed the data using thematic analysis [20]. The research team was composed of three women from different disciplinary backgrounds [allergist (SK), qualitative scientist (EA), sociologist (MS)]. The Principal Investigator (SK) had 15 years of experience with conducting penicillin allergy challenges and contributed important clinical and contextual insight to the analysis discussions.

The codebook was developed through an iterative process. The team members read interview transcripts and took notes about excerpts that fit the TDF concepts. Emerging concepts that did not fit into TDF were categorized as new codes (e.g. patient empowerment). The team members coded each transcript separately and compared their codes during biweekly team meetings to ensure codes were applied consistently and to reach consensus for each transcript. Memos created throughout analysis tracked our thoughts and findings.

We created data tables to organize the barriers within each TDF domain. For the first set of tables, we summarized the data by clinician role, tabulated by the primary TDF domain, setting (inpatient vs outpatient), and other domains co-coded for that content. Keeping track of the domains co-coded enabled us to capture of the interaction between domains. The next analytic step included consolidating the content by clinician role into one table of meta-themes. EA and MS consolidated the tables and took extensive notes of their thought processes. For the last analytic step, SK reviewed the consolidated table and finalized the themes through discussions with EA and MS.

## Results

We interviewed 20 clinicians. Participants included 3 hospitalists, 5 inpatient pharmacists, 1 infectious disease physician, 2 anti-microbial stewardship pharmacists, 4 primary care providers, 2 outpatient pharmacists, 2 resident physicians, and a nurse case manager for the allergy service. We should note that the outpatient clinicians we interviewed for this study did not participate in the previous quality improvement based initiatives at our facility. Therefore, they had not received any penicillin allergy education and were not provided access to the CDST prior to our outpatient interviews. However, given the need for future involvement of our outpatient healthcare team, we recruited them to gather preliminary data surrounding possible and perceived barriers to expanding penicillin allergy de-labeling interventions to outpatient settings.

**Fig. 2:**
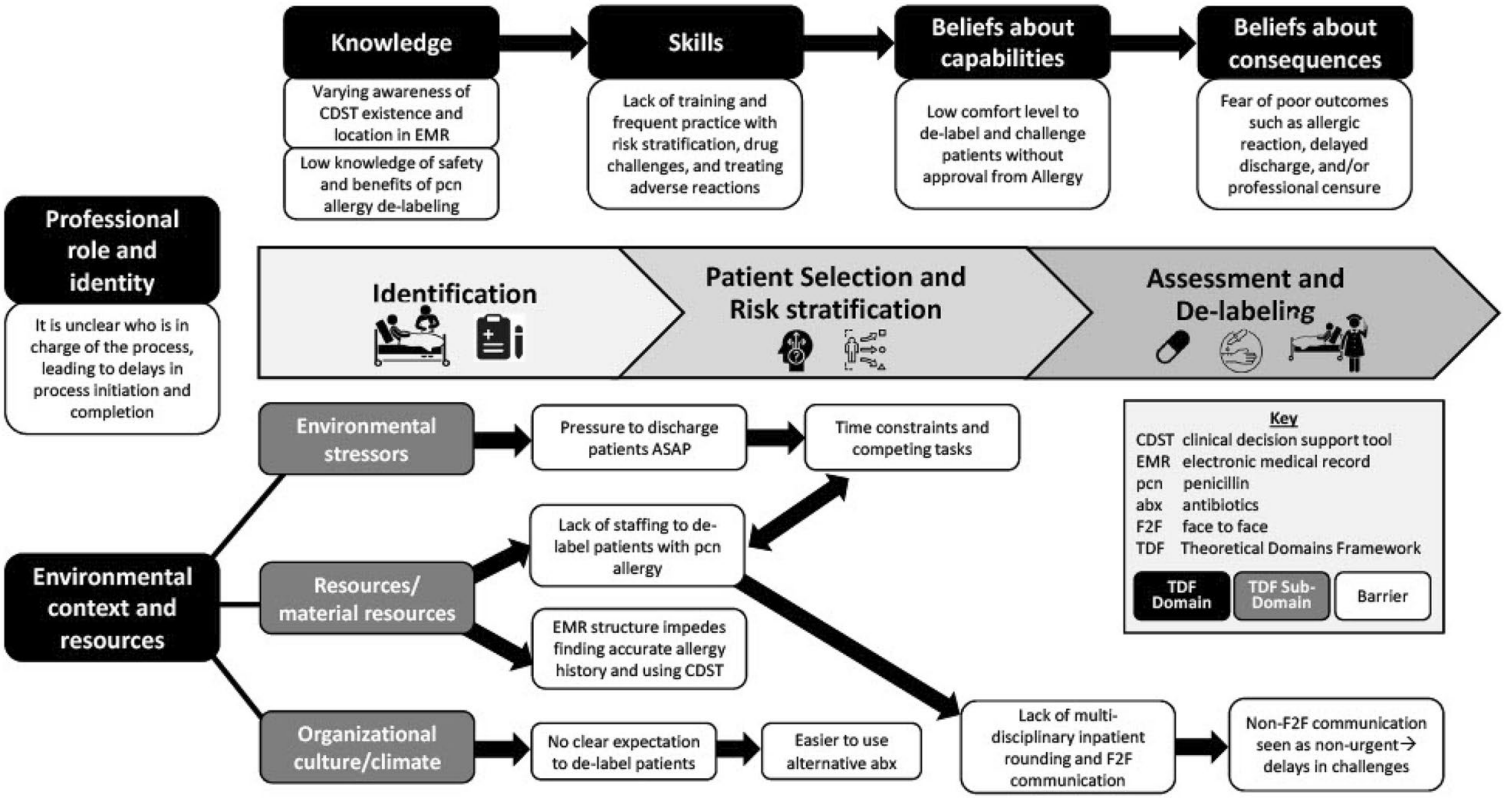
Key barriers to penicillin allergy de-labeling process for inpatients

The factors that contributed to barriers to penicillin allergy evaluation and de-labeling were classified under six TDF domains spanning both individual and system-level determinants. In our study, we found that the factors related to knowledge, skills, beliefs about capabilities, beliefs about consequences, environmental context and resources, and professional role and identity were the most prominent barriers to penicillin allergy evaluation (Fig. 2).

### Inpatient setting

#### Knowledge, skills, beliefs about capabilities and consequences

All inpatient clinician groups were aware of the scientific evidence supporting the penicillin allergy evaluation. Specifically, participants were familiar with data showing increased risk for comorbidities in patients labeled as penicillin allergic. However, they reported a need for further education on de-labeling benefits and reassurance for its safety. Health care professionals in all groups cited apprehension about inducing an allergic reaction with test dose challenges and having inadequate skills and resources to treat a possible allergic reaction. Despite this, both inpatient pharmacists and hospitalist physicians were amenable to gaining the skills to identify low-risk patients and perform test dose challenges in low-risk patients in the future. In addition, pharmacists and hospitalists felt that they needed frequent practice to maintain familiarity and comfort with the process.

When asked questions regarding using the CDST available to aid history taking, risk stratification and de-labeling of penicillin allergic patients, clinicians in all groups reported a lack of knowledge on where the CDST could be found within the EMR or how to apply it. Upon reviewing the penicillin allergy algorithm (Additional file 2: Appendix S1b), residents and pharmacists noted that the tool was straightforward. “I think [the] algorithm is really helpful. I think the biggest barrier is if [patients] don’t remember the reaction, or can’t get enough information to feel confident, but the algorithm itself is very straightforward” (Resident 1).

However, despite this positive feedback about the CDST, infrequent engagement and lack of practice with the task diminished clinicians’ beliefs about their self-efficacy to effectively participate in the penicillin de-labeling process. Infrequent engagement with de-labeling also influenced a perception that identifying suitable patients took a lot of time. Participants stated that they would need to hone their skills to navigate the EMR system and access the patient history and lists of previous and current medication lists. A lack of self-confidence in their skills with risk stratification and treating possible adverse reactions were noted as specific barriers. For example, a resident said they are not very comfortable with treating patients who may have adverse reactions during the drug challenges: “I know drips and epinephrine things. I just haven't had the opportunity to treat many patients with an acute reaction” (Resident 2).

Even though most participants acknowledged the positive consequences of removing allergy labels from patient records, the fear of erroneous de-labeling and patients having a serious allergic reaction as a consequence prevented clinicians from engaging in the de-labeling process. For example, hospitalists were worried that adverse reactions would add complexity to patients’ clinical care or extend their hospital stay. Pharmacists raised concerns regarding disciplinary action following an error. Several pharmacists noted that if there was evidence in the EMR that a patient was prescribed penicillin in the past, they felt confident to update EMR with a note that the patient tolerated penicillin in the past. However, they were still reluctant to de-label without consulting the patient’s hospitalist, primary care doctor or an allergy specialist, suggesting a lack of confidence or trust in interpreting allergy data in the EMR. “I think it is really tough to take that allergy off the chart unless the patient gets that specific antibiotic while they're here, we have the discussion with the providers that the patient tolerated it just fine and are comfortable that documenting that we're pulling it off the chart completely” (Pharmacist 5).

#### Professional role and identity

As demonstrated in Fig. 1, penicillin evaluation and de-labeling is a multidisciplinary process requiring a collaborative approach. The interdependent nature of the process requires high levels of coordination and communication within and among teams. When a patient is admitted to the hospital, the pharmacy team (either inpatient pharmacist or antimicrobial stewardship pharmacy) usually initiates the de-labeling process by identifying patients with penicillin allergy and conducting a medication reconciliation based on patient’s medical records. In the next step, the patient is risk stratified and direct drug challenge is recommended if the patient is low risk. In the current workflow, the Allergy Consult service evaluates and de-labels the patient, although ideal future workflows would empower inpatient pharmacists and hospitalists to fulfill this role for low-risk patients. Currently, pharmacists and hospitalists expressed that they needed the Allergy service’s approval to de-label a patient especially if there is disagreement among teams.

“There's sometimes a little bit of disagreement with the history taking and the one that comes up all the time is, did the patient really have hives or true urticaria? And then almost always in that situation, we default to the most conservative or safest option, [between] skin testing, getting allergy involved, or doing an oral test dose”. (Hospitalist 1)

Clinicians noted that obscurity on which teams would take the lead on de-labeling created barriers to developing robust workflows in clinic. In addition, discomfort with the tasks that did not clearly fall under a specific specialty—such as ordering of the test dose and monitoring the patient during oral challenge—discouraged clinicians from engaging in the de-labeling process. Specifically, neither pharmacists nor hospitalists felt that removing the allergy labels from patient records fell within the inpatient teams’ current professional roles. Because there was not a point person or a group who clearly championed the initiative, the task ended up “bouncing” among teams and fell through the cracks (Table 2). This, in combination with other environmental stressors, resulted in inconsistent clinical workflows and variable application of the penicillin allergy CDST.

“I guess it's a little unclear [who takes the lead on de-labeling]. Um, I think that, you know, teams, individual medical teams do try to do something. It is certainly not very systematic amongst the teams” (Hospitalist 2)

**Table 2:** Representative quotes demonstrating perspectives of inpatient and outpatient clinicians

Domain	Constructs	Quotes from inpatient clinicians	Quotes from outpatient clinicians
Knowledge	Knowledge (scientific rationale)	I think a lot of people would be convinced by the data out there about long term benefits to doing this. And people are probably not super aware of the data. So I think the data would be a good selling point overall (Resident 1)	Probably the majority of providers don't know that de-labeling of something that is actually a viable thing that we can do a lot of times historically or otherwise. (PCP 4)
	Procedural knowledge	I think a big barrier is just that we have SharePoint, we have all these other folders, there’s different sites. I'm sure I could find [the CDST] if I went to go look for it, but we have so many different places to start looking that it's just hard to find where everything is, and how updated it is, because we have old versions of stuff and new versions. Our file organization system isn't the best. (Pharm 4)	I am aware that there are kind of protocols to look at history and things like that to delabel and then a paradigm or an algorithm to go through. But I don't think the majority of primary care doctors are. (PCP 4)
Skills	Skills	In terms of evaluating their risk for an actual activation of the allergy, I wouldn't feel extremely comfortable, especially doing that on my own. I feel like it's always been a discussion with the team and then if ID needs to get involved in evaluating from that standpoint of group collaboration of what do we think, when was this reaction? …But in terms of actually assigning a risk to it, I don't feel too comfortable at this point doing that on my own. (Pharm 5)	I think I'd be somewhat worried about the volume. I could just see getting trained initially and then we do this for one patient a month and no one has a reaction for 2 years. And then someone does have a reaction and we don't feel as comfortable anymore. (PCP 3)
Beliefs about capabilities	Perceived competence	We rarely do antibiotic test doses. So there may be a lot of concerns about doing that inpatient. So I think familiarity and comfort level across the disciplines is probably one of the barriers. Again, if we do this maybe four times a year, that's really quite infrequent that we're challenging patients. (Hospitalist 2)	Without having been there in the initial moment when they had the reaction, I think it's hard sometimes to be able to, to sort of distinguish and feel confident and questioning whether it was a true allergy. PCP#?
Beliefs about consequences	Outcome expectancies	I feel like if they had penicillin allergy on their chart, but maybe you didn't think it was all that severe, so you give penicillin anyway and having them have an anaphylactic reaction and possibly bad outcome. I think that's probably my biggest fear or barrier to removing the label or giving someone penicillin when they have a documented allergy. (Resident 2)	I haven't done the direct or, the ordering provider, the administrator, the monitoring provider. So I think without experience in that, it would be relatively unsafe and then we don't have any protocols for monitoring after things outside of a few minutes after a vaccination in clinic, we don't have a structure in place to have somebody actively monitor for longer. (PCP 2)
Professional role and identity	Professional role	It’d be nice to know who is ultimately going to lead the charge, because I feel like a lot of times, we might see it first, because the pharmacy technician put it in med rec. And then if we reach out to the team, I feel like, then sometimes it gets bounced from the team to ID. And if ID recommends getting allergy involved, and it kind of seems like it's always the next person who will be looking into it. And a lot of times I feel like that's where it falls through the cracks. So, if we knew who is going to take charge of it from the beginning, because by the time all those things have happened, the patient might be ready for discharge and then this falls through the cracks anyways, they've already selected a different antibiotic and are being discharged on something else. So, just kind of knowing who ultimately is in charge of that follow up. (Pharm5)	I guess historically part of the problem has been sort of ownership of that and kind of a belief that once it's on the chart, it's gold and we're not going to re diagnose a patient or kind of delve too much into that…so unless someone is prompting us to do that, it’s not something we're necessarily going to go into. (PCP 4) I guess we would wonder what standard of care is, if it's standard for primary care to be doing this or if it's standard for allergy to be doing it…. I would just wonder if it's kind of outside the typical realm of what primary care would be doing to actually administer the trials…. I think there's a lot of very specific primary care things like healthcare maintenance type things that we don't have time to complete all of that. And so I would wonder if adding something that was more specialty driven is the best use of primary care resources but, but not impossible. (PCP 2)
Environmental context and resources	Environmental stressors	You get done with a long day at the hospital and it's like 6 pm and you're ready to go home. You could always ask yourself, could I go talk to this patient some more about penicillin allergy de-labeling? The answer is yes, there's always time there, but is there time within reasonably normal working hours that isn't going to burn the inpatient team out? (Hospitalist 1) It's hard right now, the way the model is, when you're a pharmacist, you have two medical teams essentially that you're covering. And so usually they round at the same times and so you can't be in two places at once. (Pharmacist 2)	If primary care does all the preventative care that it’s supposed to do for each patient that comes in, that's going to take seven hours out of the day. Plus all the acute care needs that patients are bringing up and things they have to address and paperwork and other things. And so eventually the day just kind of runs out of time and, you know, we kind of struggle to do the things that typically fall under the umbrella of primary care in the way the system is currently set up. (PCP 4) A lot of times when I'm seeing a new patient, there's so much to get done. There's so much medical history that when I'm entering the allergies, I'm kind of trying to go as fast as I can and it does ask, what the reaction was, but sometimes they're just like, ‘oh, I don't know.’ And I'm just like, okay ‘unknown, next.’ [Laughs] So, I think probably just general primary care time constraints is the big one. (PCP 3)
	Resources/material resources	Eventually a lot of these patients need to be sent to the allergy clinic for testing or could get tested in the hospital. And we don't have FTE either here or in the allergy clinic to do that… But we have limited ability to do that because of [Allergy’s] space, their FTE and then our FTE. So that that's probably the biggest barrier. (AMSPh1)	At our community based outpatient clinics, I don't know if I would want to do this if a patient had a reaction, so if this would be done at the main hospital where, if something did happen, we've got the emergency department, we've got inpatient services right there… I could see some hesitancy with doing this procedure in some of our community based outpatient clinics or clinics that just aren't as well supported to navigate an issue if it arose. (Outpatient Pharm 2)
	Organizational culture /climate	I think a lot of conversations now happen by Microsoft Teams. I think the lack of an in-person communication probably impacts that, like you don't want to bother them as much by sending them yet another Teams message, or your point might not necessarily get across in the electronic communication. I think a lot of times, often it’s just easier to have that face to face conversation, and really not having that with the physician teams, like I almost never see the physicians in person anymore, when I'm staffing on the floors, I guess I should say. (AMS Ph2)	For a procedure that takes 90 min, I feel like that might be a tough sell to have the team available…. that could be overwhelming if there's a lot of those coming through…. we're in a workforce shortage right now within primary care providers, LPNs, nurses… That, I think is something else to note, work availability of personnel to be able to implement it. (Outpatient Pharm 1)

#### Environmental stressors, resources, and organizational culture

Pharmacists, hospitalists, and specialty consult services described an organizational culture where workload and competing priorities prevented implementation of penicillin allergy protocols in the inpatient setting. The teams’ abilities to focus on patients who are penicillin allergic were hindered by the need to prioritize other competing quality measures and exacerbated by the limited inpatient bed availability.

“I think from an inpatient perspective, it's probably the culture that ‘we need to address the things that need to be addressed as an inpatient, and the rest can be pushed to outpatient world.’ So that tends to be a general thought process. And it's sometimes appropriate, and sometimes it isn't, and penicillin allergy falls in that bucket. So, I think that is probably something in the organizational culture". (ID MD1)

Participants also said that using an alternative antibiotic was easier than evaluating the allergy. This perception was reflected in workflows, especially in times of stress and periods of competing priorities where individuals defaulted decision making to prioritize discharging of patients. “In terms of time to evaluation and treating the patients effectively, a lot of times using an alternative antibiotic is the path of least resistance if there is an alternative there. But if we’re kind of stuck between a rock and a hard place, and we need that one antibiotic, maybe that is the way to go then. But I feel like I've just seen so far that a lot of times a different antibiotic is picked just to steer clear of that allergy for the time being” (Pharmacist 5).

With easy access to alternative antibiotics, clinicians prioritized other competing tasks and postponed de-labeling to an unspecified time or deferred to an allergist. Although allergists assumed a leadership role by becoming the point person for patients with complex histories, insufficient resources such as staffing and clinical space prevented them from consulting with all potentially eligible patients. Overall, emphasis on rapid discharge workflows interrupted the momentum and often led to patients being discharged before evaluation.

“I think de-labeling is important but right now, the hospital is completely full every day. We are getting messages on the screen, ‘discharge your patients as fast as you can.’ So, everything becomes secondary to getting the inpatient work done and getting the patients out of the hospital as quickly as we can”. (Hospitalist 1)

Team members described how the priority to discharge patients quickly predisposed them to dismiss tasks that may delay discharge. This was exacerbated by time constraints and the precedence to make beds available in case of an urgency, especially during Covid surges. The inpatient healthcare team often deferred penicillin allergy evaluation to a later, undefined future patient encounter: “We can't be here every hour. You're kind of having to pass the buck to somebody else to take care of it” (Pharmacist 3). One exception that facilitated allergy evaluation was if the penicillin allergy specifically affected the patient’s current hospital course.

The lack of adequate staffing to complete daily tasks was also a major barrier to de-labeling. Several clinicians pointed out that shortages of critical team members such as LPNs, and variable hospitalist schedules created barriers to standardizing and implementing de-labeling processes. In addition, inpatient pharmacists were co-assigned to two teams at once, which impeded following a patient through their entire hospital stay and prevented inpatients from being identified early enough in their hospital stay to allow time for an oral challenge. If a pharmacist or ID physician sent an alert to the inpatient team toward the end of a patient’s hospital stay, the team often deferred the task to a later time to avoid discharge delay.

“I think there's always an inherent time limitation, the admission pharmacy med rec isn't put on the chart sometimes for, like, 24 or 48 h after admission… By the time you hit 48 h, we're already planning to get [patients] out of the hospital at that point.” (Hospitalist 1)

Within the busy inpatient workflow process, ineffective communication systems further impeded the implementation of penicillin allergy evaluations. Specifically, the inability to quickly identify eligible patients within the EMR upon admission delayed risk stratification of the patients and subsequent decision making about whether the inpatient could be challenged and de-labeled by the inpatient team. Pharmacists, residents, and hospitalist physicians cited difficulties finding the CDST within the EMR due to the unintuitive nature of the system. Small errors such as not updating the history within the allergy field and indicating the relevant clinical encounter often buried important information in the clinical record, limiting data accessibility. Residents pointed out additional challenges with accessing patient history in the system, especially if they were accessing allergy records from a different institution.

Clinicians discussed a number of factors related to the culture of the organization. The decrease in staffing due to COVID and inpatient COVID surges resulted in siloed teams and reduced opportunities for multidisciplinary discussions. For example, pharmacists noted that they no longer rounded with the teams. Multidisciplinary communications were reduced to Teams messages, which made it harder to provide the team with recommendations about de-labeling and to initiate the process. Both pharmacists and hospitalists described how increasing reliance on asynchronous messaging led to ambiguity in recommendations and created the perception that recommendations to challenge patients were less urgent than recommendations that were given in person. Similarly, suggesting penicillin challenges through CPRS notes was considered as “noncommittal,” as notes were a passive form of communication, compared to recommendations conveyed over a phone call or in person. Hospitalists acknowledged that they did not always prioritize ID recommendations documented in CPRS.

“I think it is a much more passive form of communication of just assigning people to notes. It's very noncommittal by the signature that you've received that, whereas, you know, if you had a phone call, it may convey more importance”. (Hospitalist2)

### Inpatient to outpatient transitions

When the inpatient pharmacists and physicians could not de-label a patient during their hospital stay for reasons such as competing priorities, or workflow issues or pressures, they deferred the de-labeling tasks to outpatient care. However, outpatient pharmacists and primary care providers (PCP) in our study expressed several concerns with taking on penicillin de-labeling as a responsibility.

#### Barriers to de-labeling in primary care settings

PCPs and outpatient pharmacists echoed the barriers described by inpatient clinicians related to knowledge, skills, beliefs about capabilities, beliefs about consequences, and professional role (Table 2). Because these clinicians had not participated in previous quality improvement-based initiatives surrounding penicillin allergy de-labeling, they expressed hesitation about their level of knowledge and training surrounding risk stratification and oral challenges. They reported that they would need reassurance about the safety of the procedures through practical guidance and protocols on risk assessment, while ensuring that only low-risk patients would be de-labeled. They also expressed that even with updated training, they may still feel ill equipped to safely address patients’ potential allergic reactions during oral challenges because of infrequent practice. Several PCPs noted that assessing the accuracy of a penicillin label in patient records had not been part of their workflow in the past so they “[did] not think to assess it.” Additionally, because PCPs did not regularly assess the accuracy of penicillin allergy labels, they did not always remember to refer patients to the allergy clinic: “I think recognition is probably the biggest thing. It hasn’t been part of my workflow in the past to look for penicillin allergy and then to think to assess whether it’s real” (PCP2). While the PCPs thought they could play a role in patient identification by increasing their exploration of patients’ allergy history and referring patients to allergy for further assessment, they expressed that conducting oral challenges would fit better into a specialty role rather than primary care.

Outpatient clinicians also described barriers related to environmental stressors, organizational culture, and resources, and pointed out how those barriers would make it challenging for them to incorporate penicillin de-labeling into their workflows. In particular, they expressed that primary care already has so many other tasks they must cover in each appointment, that discussing and addressing penicillin allergy is a lower priority given their time constraints. Because “identifying low-risk patients and having them go through a 90-min test might be a tough sell to have the team available” (Outpatient Pharmacist 1), they preferred de-labeling tasks to be performed in the allergy clinic or by the inpatient team. Additionally, they felt that lack of emergency resources at community clinics to treat potential allergic reactions, lack of space to conduct challenges, and lack of support from nursing staff due to staff shortages were significant hurdles. Outpatient pharmacists also noted that the CPRS system could be “clunky,” making it difficult to find protocols and access accurate patient history.

“We're also struggling with space concerns at the facility where I work. I just don't think the building management would like to have people sitting around for 2 h when we don't have enough rooms as it is”. (PCP 1)

## Discussion

The benefits of penicillin allergy de-labeling for patients and antimicrobial stewardship has been widely reported [11, 21]. Removing inappropriate penicillin allergy labels from patient health records can facilitate prescription of first-line treatments for infections, reduce side effects, and improve recovery [22]. However, large-scale intervention studies on the essential components and barriers to establishing a replicable process for penicillin allergy de-labeling interventions have not been conclusively described [23]. For this reason, studies investigating barriers and a process for promoting implementation and sustainability of penicillin allergy de-labeling interventions can have significant impact on scaling up de-labeling initiatives.

Barriers at both the individual and system level can have profound influence on whether an intervention is successful. We found that gaps in general scientific knowledge regarding penicillin allergy and more importantly, lack of skills and infrequent practice in the key steps of penicillin allergy de-labeling prevented individuals from feeling confident engaging in gathering patient histories, risk stratifying patients and when appropriate, ordering and carrying out drug challenges. Our findings are consistent with previous studies. A study by Blumenthal et al. [24] also found that 2 of 5 inpatient practitioners reported no prior drug allergy education. They also reported that only 36% of the providers knew skin testing was a valid tool for determining penicillin allergy. Furthermore, there is evidence that clinicians had limited understanding of penicillin allergy and these knowledge gaps created a wide variation in the clinical management of penicillin-allergic patients [25]. Standardizing the approach to obtaining and documenting the drug allergy history in the EMR, and having a multi-disciplinary approach to de-labeling that proactively addresses the barriers found in this article may improve future scalability of penicillin allergy de-labeling interventions.

For some hospitalists and inpatient pharmacists in our study, the fear of making errors in risk stratification and possibility of causing an allergic reaction during challenges prevented them from participating in the intervention altogether, even for patients at low risk for allergic reactions. These individual level barriers originated from lack of confidence in determining the patient’s risk of future reaction, and discomfort managing the consequences to both the patient and healthcare team if adverse reactions developed. This finding is not surprising, as other studies showed that general practitioners were reluctant to prescribe penicillin even after a successful oral challenge [26–28]. Fragmentation of allergy-related documentation in the EMR and having easy access to second-line antibiotics also reinforced this behavior [29, 30].

Penicillin allergy de-labeling is a multidisciplinary objective without clearly defined ownership, roles, and responsibilities [31]. Almost all clinician groups in our study noted confusion around which clinical roles “own the process.” When coupled with lack of knowledge and comfort in tasks involved in penicillin de-labeling, clinicians most often revert to the pre-test labels [32]. A multidisciplinary collaboration in clinic with clear role distribution, buy-in from leadership [33], and designated champions [34] with dedicated effort to promoting and implementing de-labeling intervention is necessary. External change agents could also be appointed to deliver de-labelling interventions to ensure success in clinics [35].

Although there is evidence that a complete drug allergy history can be obtained within two minutes [36], clinician perceptions around time constraints, exacerbated by other tasks that imposed cognitive and temporal challenges, prevented them from engaging in de-labeling. These challenges included overwhelming pressure to discharge patients quickly to address inpatient bed availability. Similar concerns were discussed in previous studies [37]. Previous studies confirmed that algorithms were a safe approach to identify low-risk patients [38] and could reduce the use of broad-spectrum antibiotics as part of an antibiotic stewardship program [39]. Our results also supported that having decision support tools embedded in the EMR could alleviate perceived time pressures once the clinicians’ self-efficacy to access and utilize these tools in their workflows were improved through training.

Our results constitute the first stage of development of a multi-method, multi-stage behavioral intervention targeted to reduce barriers to de-labeling in the inpatient setting. Using TDF as our framework allowed us to elaborate on both the hospital context and the underpinnings of clinician perceptions and behaviors that ultimately hinder their engagement with de-labeling. The psychological constructs included in the TDF and the comprehensiveness of the framework [40] enabled us to capture not only the individual behaviors but also the interdependent nature of workflows that may influence the group conformity and culture. The results of our analysis demonstrate what factors influenced usage of our penicillin allergy de-labeling CDST. We also illustrated why de-labeling endeavors failed in this singular inpatient setting. By targeting these factors, which broadly fell into six specific theoretical domains (knowledge, skills, beliefs about capabilities, beliefs about consequences, professional role and identity, environmental context and resources) and ten constructs (procedural knowledge, knowledge of task environment, skills, practice, perceived competence, outcome expectancies, professional role, environmental stressors, resources, and organizational culture), we will be able to develop theory-based solutions to change professional practice and design an evidence-based, robust de-labeling intervention that will be scalable to larger contexts.

Our study has a number of limitations. Because it is a single-site study, we may not have captured all barriers that occur in larger hospitals. However, keeping the data collection focused on a single site allowed us to explore the topic from a multi-disciplinary perspective and cross-check if certain barriers were observed by all stakeholders. Another limitation was that our study was primarily completed during the Sars-CoV2 pandemic, which limited face-to-face interactions with participants and severely affected the participations of inpatient nursing due to staff shortages and turnover. However, conducting our study during the pandemic provided an opportunity to document the importance of in-person communications in the clinic. Despite these limitations, our results demonstrated that individual- and system-level barriers have significant influences on the implementation of penicillin allergy interventions. Future studies detailing the success of interventions will need to address issues of interprofessional teamwork, organizational culture and the development and maintenance of skills in the entire healthcare team. In addition, our study provided preliminary data on the perceptions and attitudes of primary care clinicians toward de-labeling interventions in the post Covid era.

## Conclusion

Clinicians recognized the importance of penicillin allergy de-labeling for patients and public health. They were open to reviewing the CDST resources and considered utilizing them in future practice. However, they cited the need for more training and access to specialty services to confirm their evaluation of patients for difficult cases.

Our study demonstrated that lack of innovation champions with dedicated time and resources was a critical barrier to move de-labeling efforts forward. Clinicians expressed reluctance in leading the de-labeling initiative due to time constraints and competing responsibilities. To overcome these barriers, we will need better role clarification in the clinic, opportunities to develop necessary skills, tailored communication systems, and dedicated resources. Future interventions will need to employ a systemic approach that addresses each of the behavioral domains influencing penicillin allergy de-labeling with both an inpatient- and outpatient-based focus.

### Supplementary Information

**Additional file 1.** **Appendix 1a**: Clinical Decision Support Tool available within the VHA CPRS electronic health record with links to additional resources and order sets.

**Additional file 2.** **Appendix 1b**: This Penicillin Allergy Pathway delineates a process for risk stratifying and evaluating patients with penicillin allergy. It is embedded as link within the computerized order entry menu within the CPRS Electronic Health record.

**Additional file 3. Appendix 1c**: This order set encompasses links to commonly used test doses for penicillin and cephalosporin allergy test doses. It also includes a nursing text order detailing the workflow for nurses to administer the test dose and coordinate ordering with pharmacy.

**Additional file 4.** **Appendix 1d**: Algorithm to treat a suspected allergic reaction if it occurs during the test dose challenge. Orders for these drugs are available within the clinical decision support tool (CDST).

**Additional file 5.** **Appendix 2**: COREQ (COnsolidated criteria for REporting Qualitative research) Checklist.

## Data Availability

The datasets analyzed during the current study are available from the corresponding author on reasonable request.

## Abbreviations

TDF: Theoretical domains framework
CDST: Clinical decision-making support tool
EMR: Electronic medical record
ID: Infectious disease
PCP: Primary care provider
