# Supplementary figures and images for: Barriers to penicillin allergy de-labeling in the inpatient and outpatient settings: a qualitative study
Source: Allergy Asthma Clin Immunol. 2023 Oct 11;19:88. doi: 10.1186/s13223-023-00842-y (PMC10568923; doi:10.1186/s13223-023-00842-y)

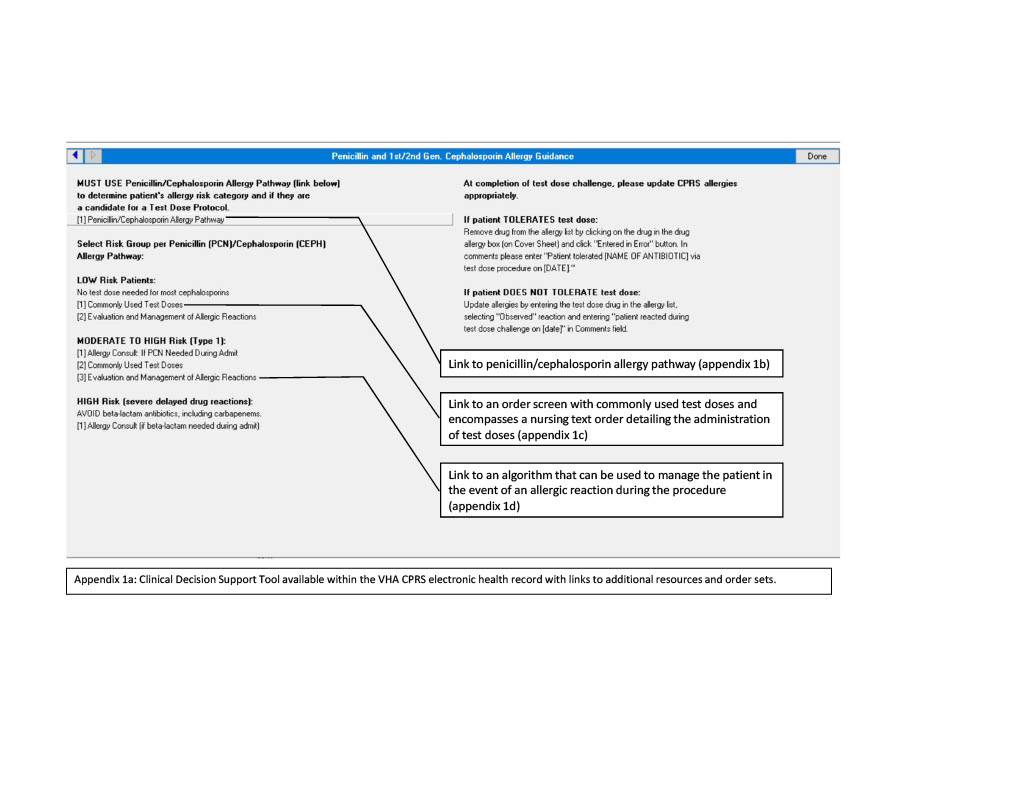

Supplement: Supplementary file 1 — Additional file 1. Appendix 1a: Clinical Decision Support Tool available within the VHA CPRS electronic health record with links to additional resources and order sets. [file 13223_2023_842_MOESM1_ESM.jpg]

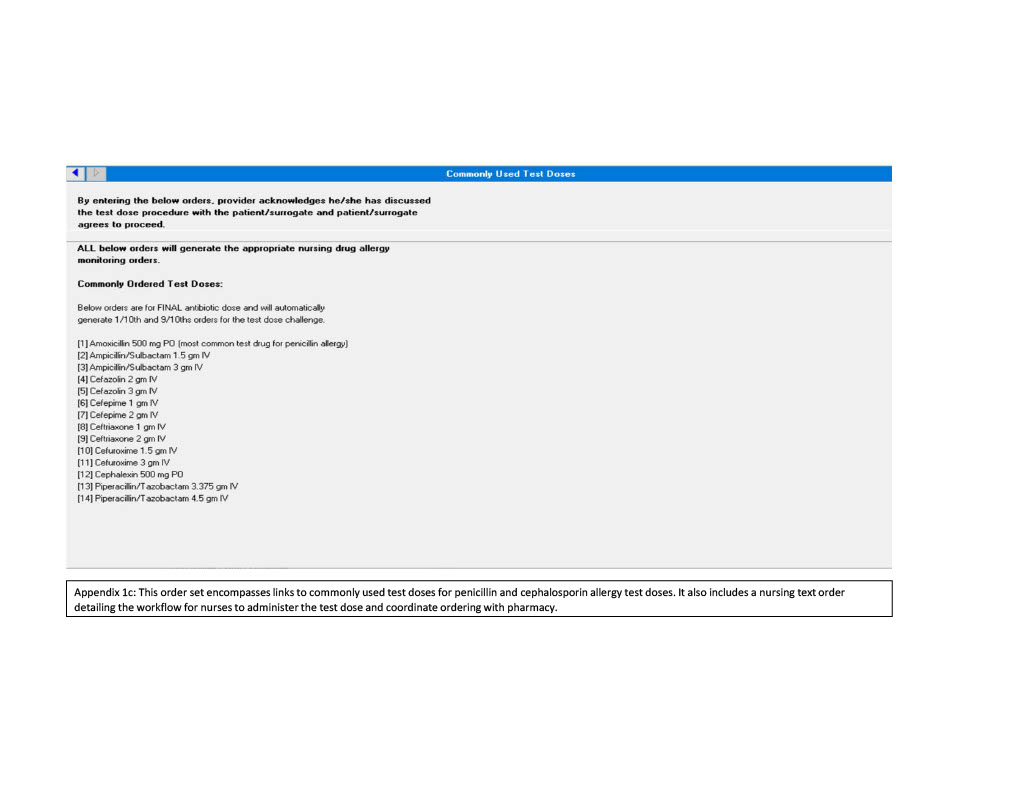

Supplement: Supplementary file 3 — Additional file 3. Appendix 1c: This order set encompasses links to commonly used test doses for penicillin and cephalosporin allergy test doses. It also includes a nursing text order detailing the workflow for nurses to administer the test dose and coordinate ordering with pharmacy. [file 13223_2023_842_MOESM3_ESM.jpg]
