# Supplementary material for: Barriers to penicillin allergy de-labeling in the inpatient and outpatient settings: a qualitative study
Source: Allergy Asthma Clin Immunol. 2023 Oct 11;19:88. doi: 10.1186/s13223-023-00842-y (PMC10568923; doi:10.1186/s13223-023-00842-y)
Supplement: Supplementary file 2 — Additional file 2. Appendix 1b: This Penicillin Allergy Pathway delineates a process for risk stratifying and evaluating patients with penicillin allergy. It is embedded as link within the computerized order entry menu within the CPRS Electronic Health record. [file 13223_2023_842_MOESM2_ESM.docx]

Penicillin Allergy Pathway

| **HIGH RISK (severe delayed drug reactions)**  Serum Sickness  Stevens-Johnson Syndrome/ Toxic Epidermal Necrolysis  Acute Interstitial Nephritis (AIN)  Drug Rash Eosinophilia with Systemic Symptoms (DRESS)  Drug induced Hepatitis  Hemolytic Anemia Drug Fever |  | **MODERATE TO HIGH RISK (Type 1)**  Anaphylaxis  **OR**  Hives (pruritic and welt like rash occurring in rapid onset after penicillin drug administration)  **OR**  Angioedema (related to penicillin drug exposure) |  | **LOW RISK**  Rash (fixed drug eruption or maculopapular rash) without systemic or high-risk features*  **OR**  Remote reaction (>10 years ago) without systemic or high-risk features*  **OR**  Medical Record lists allergy but patient denies history of allergy |
| --- | --- | --- | --- | --- |
|  |  |  |  |  |
| Patients with Type II-IV are not candidates for skin testing or test dose challenges and avoid using Penicillins, Cephalosporins or Carbapenems, use alternative agents by microbial coverage  If Penicillin/Cephalosporin is clinically indicated, consult Allergy and/or Infectious Disease |  | **PREFERRED:** Cefazolin**, Cefuroxime, 3^rd^/4^th^/5^th^ generation Cephalosporins via test dose procedure***  Alternatives:  Carbapenems via test dose procedure*** (less preferred due to excessively broad spectrum)  OR  Aztreonam (requires restricted drug consult)  OR  Alternative non-beta lactam by microbial coverage  OR  If a penicillin or non-cefazolin, non-cefuroxime 1^st^ or 2^nd^ generation cephalosporin is preferred, consult Allergy |  | **PREFERRED:** Cefazolin**, Cefuroxime, 3^rd^/4^th^/5^th^ generation Cephalosporins at normal therapeutic dose  **IF A DIFFERENT BETA LACTAM IS PREFERRED OR IF DE-LABELING PENICILLIN ALLERGY IS DESIRED, COULD CONSIDER:**  Challenge via Test Dose Procedure to β-lactams using the order set found under: Ward Order Menu 🡺 Antimicrobial Decision Support 🡺 Penicillin Allergy Guidance  ***If patient passes challenge, REMOVE penicillin allergy from the allergy list by right clicking the allergy and choose***  ***“Mark selected allergy as entered in error”***  Alternatives:  Carbapenems at therapeutic dose (less preferred due to excessively broad spectrum)  OR  May use non-cefazolin, non-cefuroxime 1^st^ or 2^nd^  generation cephalosporins via  test dose procedure*** |

*High-risk features include mucosal involvement, severe cutaneous reactions, nephritis, hepatitis, eosinophilia, cardiopulmonary compromise

**Cefazolin is structurally unique from other 1st generation cephalosporins. Risk of cross reactivity with patients with penicillin allergy is less than 0.2-0.7%, compared to 2-7% for the other 1st generation cephalosporins.

*****Test Dose Procedure:**

Physician to discuss the Test Dose strategy with the patient

Physician to order 1/10^th^ of the desired medication

as well as

9/10^th^ of medication (two separate orders)

Several pre-built test dose beta lactams are available on the following CPRS menu: Ward Order Menu 🡺 Antimicrobial Decision Support 🡺 Penicillin Allergy Guidance.

Physician to remove Allergy listing in CPRS if test dose tolerated. Nursing staff:

Administer 1/10^th^ the dose of the desired medication

Observe for 30 mins: symptom check at 15 mins and at 30 mins Administer the remaining 9/10^th^ of the dose

Observe for 60 mins: symptom check at 20 mins, 40 mins and 60 mins.

If patient symptomatic, contact medical team and document in provider contact note. If patient not symptomatic, inform Physician team that the test dose was uneventful.

Appendix 1b: This Penicillin Allergy Pathway delineates a process for risk stratifying and evaluating patients with penicillin allergy. It is embedded as link within the computerized order entry menu within the CPRS Electronic Health record
