# Supplementary material for: Barriers to penicillin allergy de-labeling in the inpatient and outpatient settings: a qualitative study
Source: Allergy Asthma Clin Immunol. 2023 Oct 11;19:88. doi: 10.1186/s13223-023-00842-y (PMC10568923; doi:10.1186/s13223-023-00842-y)
Supplement: Supplementary file 4 — Additional file 4. Appendix 1d: Algorithm to treat a suspected allergic reaction if it occurs during the test dose challenge. Orders for these drugs are available within the clinical decision support tool (CDST). [file 13223_2023_842_MOESM4_ESM.docx]

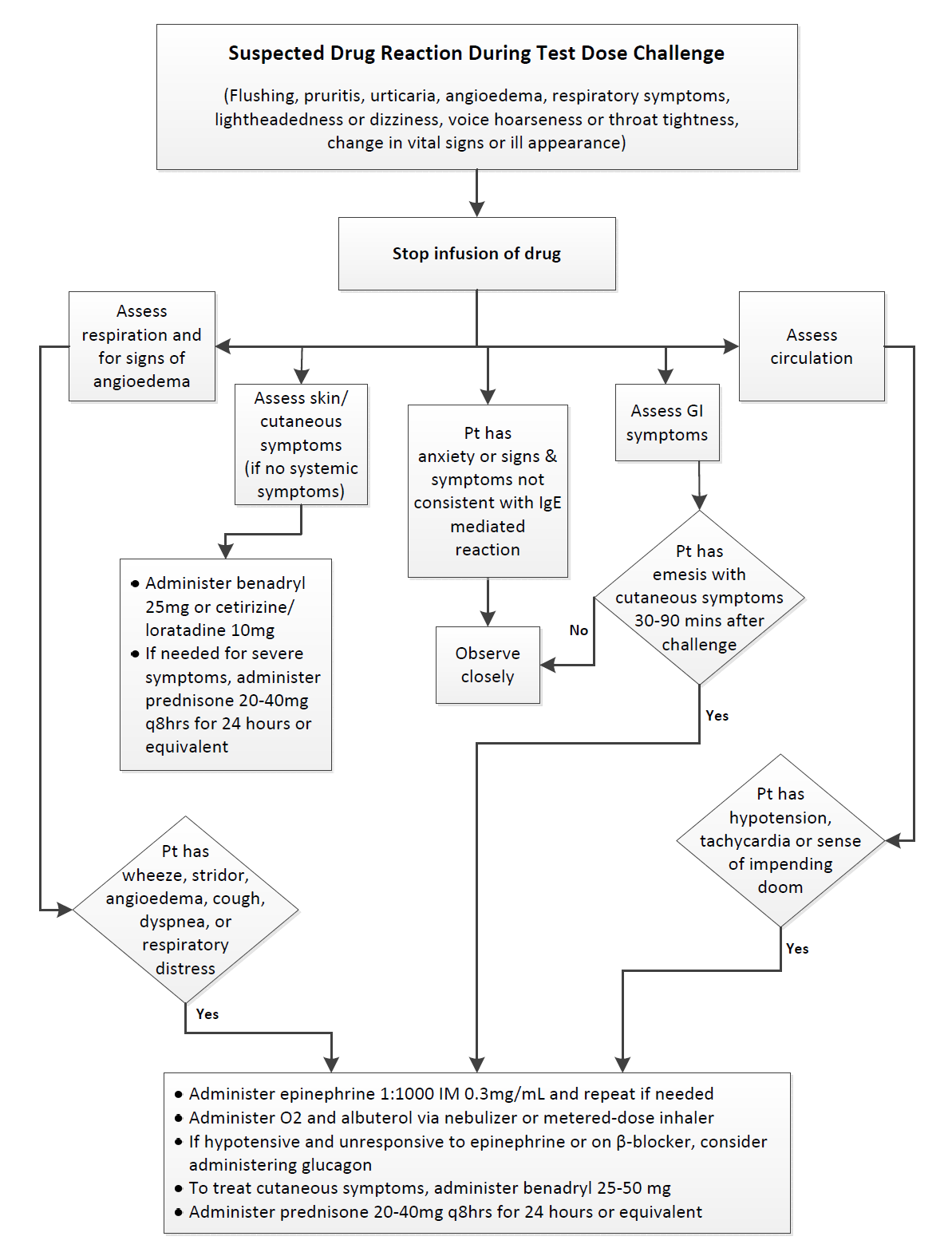


Appendix 1d: Algorithm to treat a suspected allergic reaction if it occurs during the test dose challenge. Orders for these drugs are available within the clinical decision support tool (CDST)
